# Investigating the state dependence of drug binding in hERG channels using a trapped-open channel phenotype

**DOI:** 10.1038/s41598-018-23346-x

**Published:** 2018-03-21

**Authors:** Samrat Thouta, Garman Lo, Lukas Grajauskas, Tom Claydon

**Affiliations:** Department of Biomedical Physiology and Kinesiology, Simon Fraser University, Burnaby, B.C. Canada

## Abstract

The hERG channel is a key player in repolarization of the cardiac action potential. Pharmacological blockade of hERG channels depletes the cardiac repolarization reserve, increasing the risk of cardiac arrhythmias. The promiscuous nature of drug interactions with hERG presents a therapeutic challenge for drug design and development. Despite considerable effort, the mechanisms of drug binding remain incompletely understood. One proposed mechanism is that high-affinity drug binding preferentially occurs when channels are in the inactivated state. However, this has been difficult to test, since inactivation is rapid in hERG and access to the drug binding site is limited by slower opening of the activation gate. Here, we have directly assessed the role of inactivation in cisparide and terfenadine drug binding in mutant (I663P) hERG channels where the activation gate is trapped-open. We firstly demonstrate the utility of this approach by showing that inactivation, ion selectivity and high affinity drug binding are preserved in I663P mutant channels. We then assess the role of inactivation by applying cisapride and terfenadine at different membrane voltages, which induce varying degrees of inactivation. We show that the extent of block does not correlate with the extent of inactivation. These data suggest that inactivation is not a major determinant of cisapride or terfenadine binding in hERG channels.

## Introduction

The human-*ether-a-go-go*-related gene (hERG) encodes the pore-forming α-subunit of the voltage-gated K^+^ (Kv) channel that underlies the cardiac rapid delayed rectifier current, I_Kr_. This current is essential for normal cardiac electrical activity and rhythm^1,2^. Distinct from other Kv channels, hERG channels activate and deactivate slowly, but inactivate and recover from inactivation rapidly. These unusual gating properties afford hERG channels a critical role in the repolarization of the cardiac action potential and termination of excitability^3,4^. Inherited mutations in, or pharmacological blockade of, hERG channels deplete the cardiac repolarization reserve and prolong the duration of the action potential leading to long QT syndrome type 2 (LQT2), a potentially life threatening ventricular repolarization disorder. Individuals with LQT2 have increased vulnerability to arrhythmias, such as *torsade de pointes*, which can degenerate into ventricular fibrillation and cause sudden cardiac death^1,5^. Compared to the congenital form, acquired LQT2 is an unwanted side effect of many unrelated classes of pharmaceutical compounds^6–8^. The number and diversity of drugs that can induce LQT2 through block of hERG is significant and several drugs from different classes have been withdrawn from the market because of LQT2 liability. Development of new and safe medications requires routine hERG screening and it has been estimated that ~60% of new molecules that are developed for potential therapeutic use block hERG channels^9^. Therefore, advances in understanding the structural and molecular basis of the high sensitivity of hERG channels to a wide group of drugs is needed.

The high sensitivity of hERG channels towards a diverse range of compounds suggests that they possess an unusual drug-binding site compared to other Kv channels^6^. It is well established that the majority of drugs that block hERG channels bind within the central cavity of the pore region and that channel opening is required for binding^7,10^. Early structural modelling of the hERG pore region based on the closed-state of KcsA channels highlighted the presence of two aromatic residues (Y652 and F656) in S6, unique in hERG channels, which were subsequently shown to be crucial for high-affinity drug block^6^. Drug affinity is dependent upon the hydrophobicity and charge of the residue introduced in place of Y652 or F656 leading to the suggestion that these residues interact with drugs through cation-π interactions or π-stacking interactions. There is also evidence that upon pore opening the aromatic residues Y652 and F656 rotate to face towards the central cavity of the channel^11–13^. Recently, the high resolution cryo-EM structure of the open hERG channel state revealed unusual features of the inner cavity that may provide significant insight into the susceptibility of hERG to a wide range of drugs. These include the presence of four hydrophobic pockets, each potentially capable of accommodating a drug molecule, which connect to the pore cavity just below the selectivity filter, converging at a region with strong negative electrostatic potential. These unique features may underlie or contribute to the high sensitivity of hERG channels to drug block^14^.

While the influence of gating on these recently revealed features of the hERG inner pore architecture is not yet known, there has been considerable previous debate regarding the role of inactivation in determining binding of drugs in hERG channels. Inactivation in hERG channels has a characteristic strong voltage-dependence that has been proposed to contribute to high-affinity drug binding. While a number of studies suggest that binding of high affinity blockers is enhanced by transitions into the inactivated state^15–19^, evidence suggests that low affinity blockers may not require inactivation^19,20^. Moreover high affinity drug binding does not always correlate directly with the extent of loss of inactivation induced by mutations or changes in external K^+ ^^6,7,21–26^. These studies highlight the controversy over the role of inactivation gating in determining high affinity drug binding and the need for direct approaches to assess the state-dependence of drug interactions with hERG channels.

We recently demonstrated that proline substitutions in the S6 helix impede closure of the intracellular gate of hERG channels, trapping the gate in the open state^27^. Here, we have utilized trapped-open hERG channels as a novel paradigm with which to study the role of inactivation in drug binding. hERG I663P channels are trapped in the open state over a wide range of voltages yet exhibit voltage-dependent inactivation that is similar to WT channels. This allows us to overcome a typical challenge in studying the inactivation-dependence of drug binding, which is that the kinetics of inactivation are an order of magnitude faster than those of activation, yet drugs require the intracellular pore gate to open to access their binding site. This approach enables scrutiny of the inactivation-dependence of drug binding in hERG channels by altering the membrane voltage in the same channels, rather than the more typical approach of comparing drug affinity between WT and channels in which inactivation is removed by introduction of a mutation. We compared the extent of block of hERG channels by classical blockers at different voltages, which induce varying degrees of inactivation and show that the extent of block does not correlate with inactivation.

## Results

### Inactivation is preserved in hERG I663P channels

**Figure 1:**
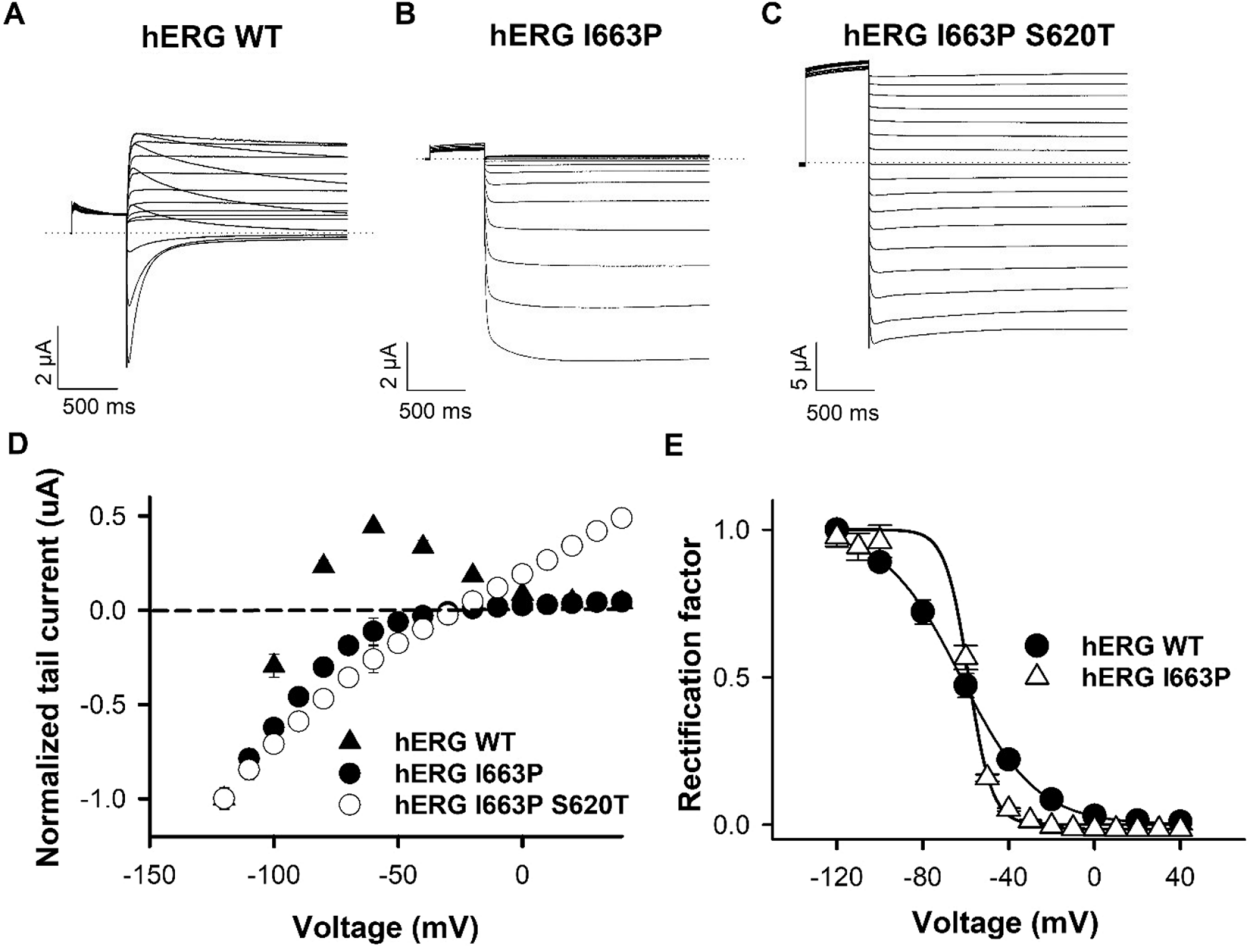
hERG I663P trapped open channels exhibit inactivation that is similar to that in WT channels. (**A**–**C**) Typical hERG WT, I663P and I663P/S620T current traces evoked during 4 s repolarizing voltage steps from +40 to −120 mV applied following a 500 ms step to +60 mV to activate channels (holding potential was −80 mV for WT and −30 mV for I663P and I663P/S620T). (**D**) Fully activated hERG WT, I663P and I663P/S620T I-V relations constructed from peak tail currents. Current amplitudes were normalized in order to compare rectification in the two constructs. (**E**) Voltage-dependence of inactivation of hERG WT and I663P channels. Rectification factor was calculated (see Materials and Methods) from the fully activated tail current data in (**D**). Data were fitted with a Boltzmann function. *V*_*1/2*_ and *k* values were −62 ± 3.1 and 17 ± 0.4 mV for WT (n = 6), and −58.6 ± 0.5 and 5.4 ± 0.5 mV for I663P (n = 6), respectively.

We have previously shown that the I663P mutation produces constitutively active hERG channels that do not appear to deactivate^27^. Comparison of Fig. 1A,B demonstrates the inability of hERG I663P mutant channels to close even during strongly hyperpolarized voltages. Typical hERG WT and I663P tail currents recorded in response to steps to different voltages following a 500 ms pulse to +60 mV to maximally activate channels are shown. In Fig. 1D, the peak tail current amplitude is plotted as a function of test voltage. These data show that, despite being trapped in the open state, I663P mutant channels exhibit strong rectification properties, consistent with hERG WT channels. Interestingly, I663P trapped-open mutant channels retain inactivation properties that are similar to those in WT channels. This can be demonstrated by comparing the extent of rectification in WT and I663P channels and calculating the rectification factor (Fig. 1E; see Materials and Methods) from fully activated tail currents (Fig. 1D). The dependence of rectification upon voltage (Fig. 1E) was described using a Boltzmann function, which yielded similar voltage dependencies of rectification in the WT and mutant channels: the *V*_*1/2*_ of hERG WT rectification was −62 ± 3.1 mV (n = 6) compared with −58.6 ± 0.5 mV (n = 6, NS, *t*-test) in hERG I663P (although the slope factor, *k*, was altered (P < 0.05, *t*-test): *k* was 17.0 ± 0.4 and 5.4 ± 0.5 mV, respectively; see Discussion section). In WT hERG channels, rectification can be abolished by the outer pore S620T mutation, which inhibits inactivation^18^. Figure 1C,D show that the S620T mutation also largely abolishes rectification in hERG I663P channels. Taken together, the data in Fig. 1 show that the I663P mutation traps channels in the open state, but preserves voltage-dependent inactivation gating.

### Ion selectivity is preserved in hERG I663P channels

**Figure 2:**
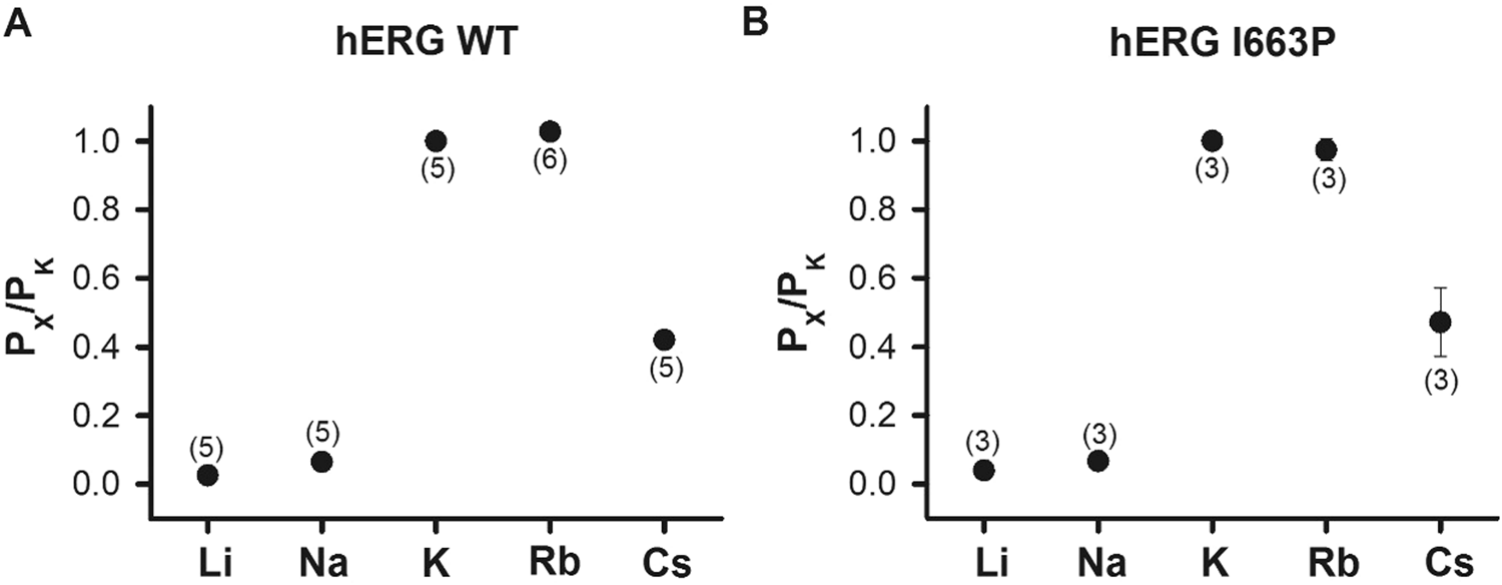
WT-like ion selectivity is preserved in I663P trapped open channels. (**A** and **B**) Plot of the permeability ratio (P_X_/P_K_) for each ion (see Materials and Methods section) in hERG WT (**A**) and I663P (**B**) channels. P_X_/P_K_ ratios for WT were: P_Li_/P_K_ = 0.02 ± 0.002; P_Na_/P_K_ = 0.06 ± 0.01; P_Rb_/P_K_ = 1.2 ± 0.01; and P_Cs_/P_K_ = 0.42 ± 0.01 (n = 5). P_X_/P_K_ ratios for I663P were: P_Li_/P_K_ = 0.03 ± 0.004; P_Na_/P_K_ = 0.06 ± 0.01; P_Rb_/P_K_ = 0.97 ± 0.03; and P_Cs_/P_K_ = 0.47 ± 0.09 (n = 3). n numbers are shown in parenthesis. The error bars are displayed behind the data points and some are so small that they are not visible.

Figure 2 shows the results of ion substitution experiments designed to measure the permeability ratio (P_X_/P_K_) of WT and I663P mutant channels. Peak inward tail currents were recorded during repolarization from −110 mV to +60 mV following a depolarizing pulse to +60 mV in the presence of external solution containing 99 mM LiCl, NaCl, KCl, RbCl, or CsCl. The P_X_/P_K_ ratio was calculated from the measured reversal potentials (see Materials and Methods). Figure 2 shows plots of the permeability ratios measured for the different ions in WT and I663P mutant channels. There was little difference between the permeability ratios of ions in the two channels. These data demonstrate that, as with voltage-dependent inactivation gating, ion selectivity is preserved in trapped-open I663P channels.

### High-affinity binding of cisapride and terfenadine is preserved in hERG I663P channels

**Figure 3:**
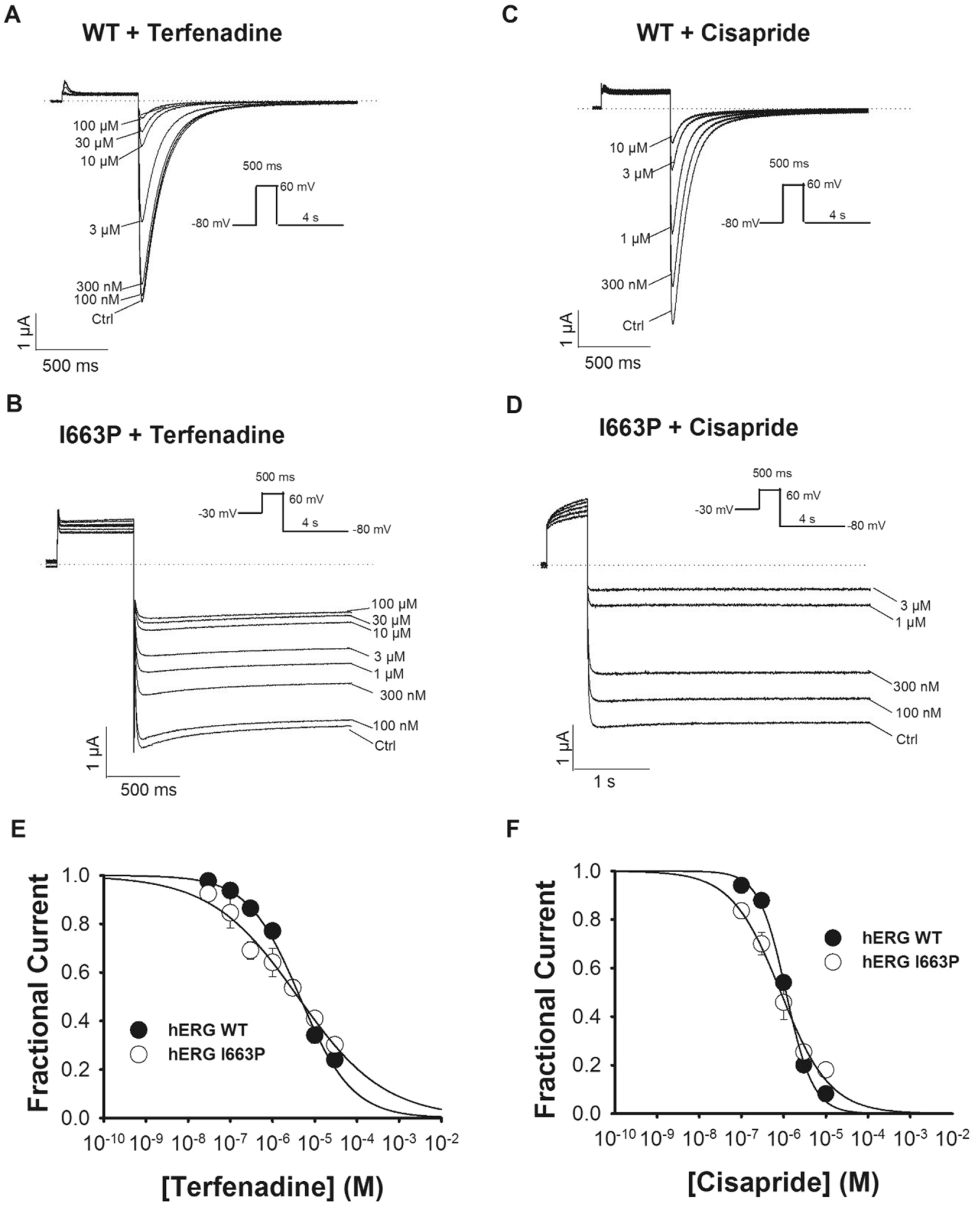
High affinity block by terfenadine and cisapride is preserved in I663P trapped open channels. (**A**–**D**) Typical WT and I663P currents recorded with 30 mM external K^+^ evoked during repetitive application of the voltage protocol shown in the insets and in the presence of the indicated concentration of terfenadine (**A** and **B**) or cisapride (**C** and **D**). The pulse frequency was 0.14 Hz, such that channels were held at the holding potential for 1 s between successive sweeps. In both WT and I663P channels, peak tail current was inhibited in a concentration dependent manner. Traces shown represent steady-state conditions at each concentration. (**E** and **F**) Concentration-effect relationship for block of WT and I663P channels by terfenadine (**E**) and cisapride (**F**), plotted from peak tail current amplitudes recorded in experiments such as in (**A**–**D**). Peak tail current amplitudes in the presence of drug were normalized to peak tail currents in the absence of drug. Fits of WT data to the Hill equation (see Material and Methods) yielded values for *IC*_50_ and *n* of: 4.4 ± 3.0 µM and 0.7 ± 0.04 for terfenadine block (n = 5); 1.1 ± 2.0 µM and 1.3 ± 0.09 for cisapride block (n = 5). Fits of I663P data yielded equivalent values of: 4.0 ± 4.0 µM and 0.4 ± 0.03 for terfenadine block (n = 4); 0.85 ± 0.2 µM and 0.7 ± 0.05 for cisapride block (n = 4).

Since I663P mutant channels are trapped in the open state over a wide range of potentials, yet exhibit voltage-dependent inactivation that is similar to WT channels (Fig. 1), we reasoned that I663P channels could be used to assess the role of inactivation in drug binding in hERG channels. Figure 3 shows that hERG I663P channels bind well-characterized hERG blockers, terfenadine and cisapride, with a concentration-response that is similar to that in WT channels. Figure 3A–D show example current traces from an experiment in which WT and I663P channels were repeatedly activated in the presence of increasing concentrations of the hERG channel blocker terfenadine (A and B) or cisapride (C and D). Peak tail current was inhibited in both WT and I663P channels in a concentration-dependent manner. This is seen clearly in Fig. 3E,F, which plots the applied concentration of terfenadine (Fig. 3E) and cisapride (Fig. 3F) drugs against mean tail current amplitude and shows fits of the data using the Hill equation. The *IC*_50_ values for block by terfenadine were 4.0 ± 4.0 and 4.4 ± 3.0 µM in I663P (n = 4) and WT (n = 5) channels, respectively (NS, *t*-test). The *IC*_50_ values for block by cisapride were 0.85 ± 0.2 and 1.1 ± 2.0 µM in I663P (n = 4) and WT (n = 5) channels, respectively (NS, *t*-test). These data demonstrate that I663P mutant channels retain high-affinity binding of hERG channel blockers. Interestingly, the Hill coefficient was subtly different in the I663P mutant channels: n values for block by terfenadine were 0.7 ± 0.04 and 0.4 ± 0.03 in WT and I663P channels respectively (P < 0.05, *t*-test). n values for block by cisapride were 1.3 ± 0.09 and 0.7 ± 0.05 in WT and I663P channels respectively (P < 0.05, *t*-test).

### hERG I663P channels did not show a strong dependence of cisapride and terfenadine binding upon voltage

**Figure 4:**
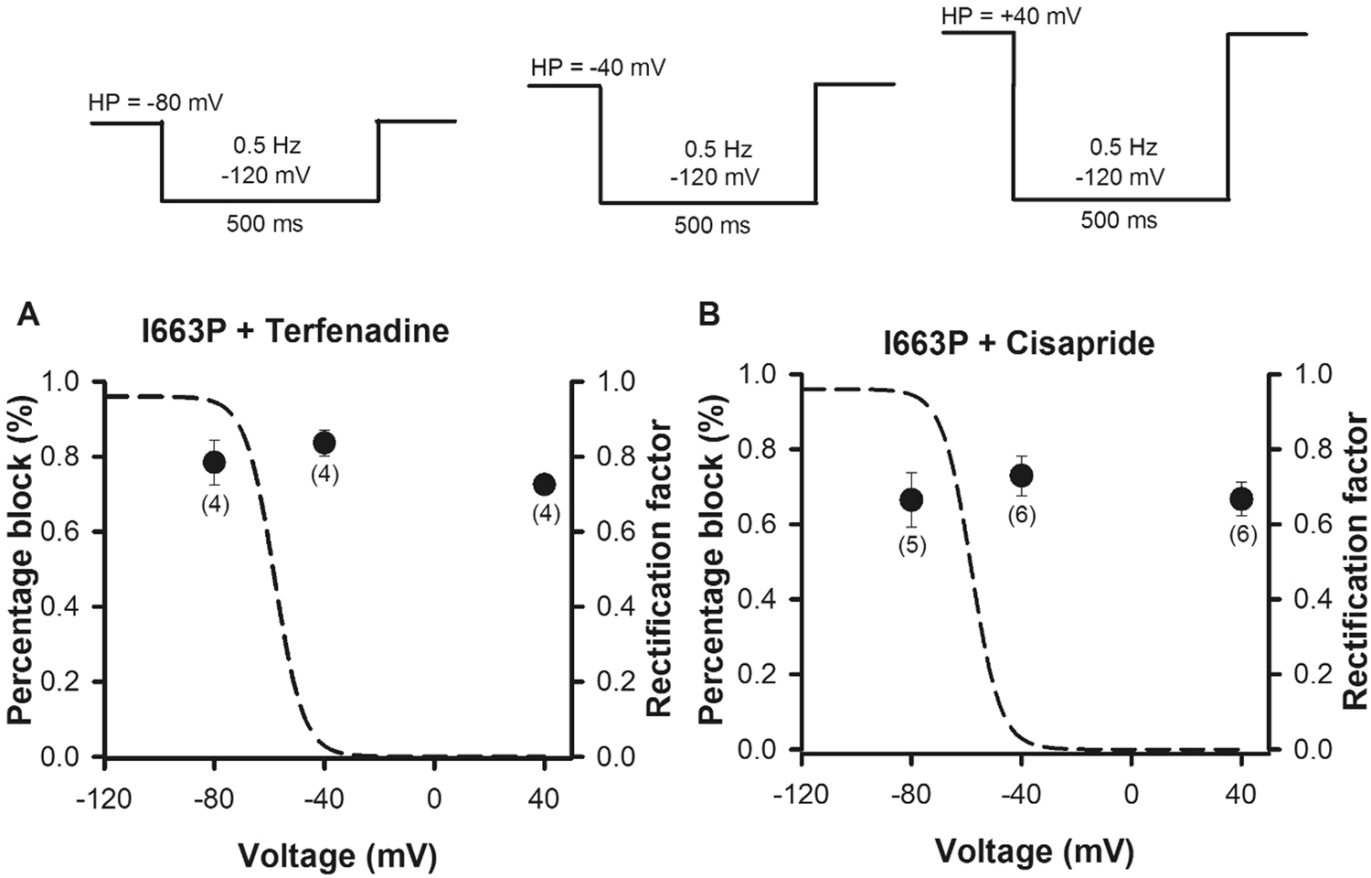
State-dependence of drug binding in trapped open hERG I663P channels. (**A** and **B**) Plots of percentage block by terfenadine (**A**) or cisapride (**B**) versus membrane voltage for I663P channels collected using the protocols shown. Pulse frequency was 0.5 Hz, such that channels were held at the holding potential indicated for 1.5 s between sweeps. n numbers are shown in parenthesis. Dashed lines in (**A** and **B**) represent the rectification factor calculated from hERG I663P fully activated currents (Fig. 1E).

Having established that voltage-dependent inactivation, selectivity and high affinity block by terfenadine and cisapride is preserved in I663P channels, we next investigated the inactivation state-dependence of drug binding. Using I663P channels enables us to scrutinize the ability of drugs to bind to channels residing in inactivated and non-inactivated states by adjusting the membrane voltage, without confounding factors, such as slow activation or the need to manipulate inactivation through the introduction of mutations. We compared the binding affinities of terfenadine and cisapride at voltages where inactivation is minimal (e.g. −80 mV) with voltages where inactivation is maximal (e.g. +40 mV). Figure 4 shows how block by terfenadine (A) and cisarpride (B) was affected by the extent of inactivation. We used a concentration that was close to the measured *IC*_50_ values (Fig. 3) so as to readily observe changes in drug binding affinity. To measure block, the drug in question was perfused whilst the membrane voltage was held at −80, −40 or +40 mV and short 500 ms test steps to −120 mV were applied at 0.5 Hz to assess channel availability. Figure 4A,B shows a plot of the percentage block at each holding potential. In the case of both terfenadine and cisapride there was no strong dependence of binding upon voltage, and each blocked significantly at −80 mV (NS, ANOVA). Figure 4A,B also show the rectification factor (dashed line) calculated from hERG I663P fully activated currents (from Fig. 2E) as a measure of the voltage dependence of inactivation to allow direct evaluation with drug block. It is clear that the extent of block by both terfenadine and cisapride does not correlate with the voltage-dependence of inactivation.

## Discussion

Since the discovery of hERG channels, significant interest has been generated due to the promiscuous nature by which the channel binds a wide variety of drugs with high affinity. Drug binding decreases hERG channel function resulting in delayed repolarization of the cardiac action potential. This can cause LQT2 syndrome and predispose individuals to cardiac arrhythmia and sudden death. The pharmacology of hERG channels is complex and incredibly interesting, and despite considerable research, the mechanisms underlying drug binding in hERG channels remain incompletely understood. It is well established that most drugs that block hERG do so once channels have activated^28,29^. For example, D540K mutant hERG channels, which show unusual opening at hyperpolarizing voltages, have revealed that, drugs are trapped behind a closed intracellular pore gate, which can dictate unbinding of the drug^10,30,31^. Above the activation gate, site-directed mutagenesis studies have identified key residues within the pore region that are required for high-affinity block of hERG channels: F557 in the S5 helix, Y652 and F656 in the S6 helix, as well as T623, S624 and V625 at the bottom of the selectivity filter^6,20,32,33^. However, individual drugs may each be coordinated in a unique way, since, for example, V625 is not required for cisapride or terfenadine block^34^. The binding of many of the drugs that have been studied is influenced by mutation of Y652 and F656, and it has been suggested that these residue side-chains form cation-π or π–stacking interactions with drugs within the pore cavity^12^, although recent evidence has challenged this idea^35^. Data based on a range of drugs and mutagenic substitutions^12^, suggests that interactions with Y652 may be more important for high affinity binding than F656, mutation of which caused the largest effect on binding affinity when channel gating was significantly altered. This suggests that allosteric factors may contribute to drug binding. Indeed, the vertical position of Y652 within the S6 helix, in particular, strongly influences drug affinity^12^ as does the relative position of Y652 and F656^36^.

The role of inactivation gating in high affinity drug binding is one of the most intriguing features of hERG channels. For example, *eag* channels, which are closely related to hERG channels, do not inactivate and are less sensitive to drug block. Mutations that introduce inactivation into *eag* increase sensitivity to drug binding^15,16,18^. Other studies have also shown reduced high affinity binding in hERG mutant channels, which either reduce (e.g. S631A, N588K, and N588E) or abolish inactivation (e.g. S620T,G628C + S631C)^15,16,19,21,37^. However, mutations that enhance inactivation, such as G648A, F627Y and S641A, showed reduced affinity for drug block by the anti-arrhythmic drug MK-499^6^. In addition, different mutations that either accelerated or abolished inactivation showed no change in the IC_50_ for cocaine block^26^, and the reduction in binding affinity of, for example, cisapride, sotalol and E-4031, does not always correlate with the loss of inactivation^6,7,21–24^. Based upon the observation that the dependence of binding on the presence of S6 aromatic residues was decreased when inactivation was reduced by the S631A mutation^20^, a model was proposed in which high affinity drug coordination requires conformational rearrangements associated with inactivation in addition to opening of the hERG activation gate^20^. In support of this model, vertical displacement of Y652 and F656 was shown to influence binding in hERG and to introduce high affinity binding of cisapride in *eag* channels in a manner that does not correlate with inactivation^11^. The observation that cisapride block of hERG loses its dependence upon the extent of inactivation when F656 is mutated, or when internal K^+^ was substituted for Cs^+^, has added to this model to suggest that inactivation gating reconfigures the position of F656 to one that is conducive to high affinity binding of cisapride, and that the internal permeant ion influences this repositioning^38^. More recently, an alternate model has been proposed that is derived from studies of concatemeric hERG channels in which the extent of inactivation can be titrated. In these studies, block by cisapride, MK-499 and dofetilide did not correlate with the extent of disruption of inactivation induced by increasing numbers of S620T or S631A mutations into the channel tetramer^23^. These findings have led to the suggestion that the S620T and S631A mutations may allosterically alter the position of the S6 aromatic residues such that high affinity binding is deterred in a manner that is independent of changes to inactivation^23^.

One of the specific challenges to studying the role of inactivation in drug binding is that inactivation is very rapid in hERG channels and access to the drug binding site is limited by slower opening of the activation gate. In addition, as described above, manipulation of inactivation by introducing mutations may alter drug binding independent of any effect of the mutation on gating. Here, we have compared cisapride and terfenadine binding with and without inactivation in the same channels, rather than between WT (inactivation-intact) and mutant (inactivation-deficient) channels (Fig. 4). We demonstrate that voltage-dependent inactivation and selectivity are preserved in I663P mutant channels (Figs 1 and 2) in which the activation gate is trapped open allowing us to directly assess the role of inactivation in drug binding simply by altering the membrane voltage (Fig. 4). A caveat to this model is that the slope of the voltage-dependence of inactivation in hERG I663P channels was steeper than that observed in WT channels (Fig. 1E), which suggests that the voltage-sensitivity of inactivation may be enhanced in trapped-open channels. Further investigation is needed to describe this since hERG channel inactivation is intrinsically voltage-dependent, i.e. not coupled to the voltage-dependence of activation^39^ and the underlying mechanism is poorly understood. However, in the context of this study, the steeper dependence of inactivation upon voltage in I663P channels would be expected to exaggerate any dependence of drug affinity on the membrane potential, yet no such dependence was observed. Instead, the data show that cisapride and terfenadine affinity were not different when channels were held at −80 mV, where channels are not likely inactivated, from when held at +40 mV, where channels occupy inactivated states. This finding demonstrates that binding of terfenadine and cisapride occurs independent of an inactivated conformation of the selectivity filter. It is interesting that both cisapride, a drug that is not trapped by the closed activation gate, and terfenadine, which is trapped, both showed a lack of dependence on inactivation. This suggests that inactivation may not be a requirement for high affinity blockers regardless of whether they are trapped behind the activation gate or not.

Our demonstration that ion selectivity in I663P trapped open channels is WT-like is important given that a number of studies suggest that the permeant ion dictates drug binding in hERG channels^22,24,40^. External [K^+^]-dependency of E-4031, quinidine and cisapride block has been documented^24^, and altering external Na^+^, Rb^+^ and Cs^+^ have also been shown to influence drug affinity^22,25,40^. Together, these findings have led to the suggestion that raising extracellular permeant ions destabilizes or knocks off the drug from its binding site^22,40^. The reduction in binding of E-4031, cisapride and quinidine with elevated external K^+^ has been suggested to occur independently of the inhibition of inactivation that is caused by raising K^+^, suggesting that the permeant ion plays a key role^22,24^. Consistent with this, substitution of internal K^+^ with Cs^+^ reduced drug binding affinity in inactivation-deficient channels 4-fold^38^. Interestingly, external K^+^ has been suggested to destabilize blockers that are not trapped by channel closure, e.g. cisapride and quinidine, while blockers that are trapped, such as terfenadine, were shown to have little dependence on extracellular K^+ ^^22^. In the current study an elevated external K^+^ concentration was used (30 mM) to ensure accurate measurement of channel current during drug block. With 30 mM external K^+^, cisapride blocked WT channels with and IC_50_ of 1.1 μM, which is similar to that reported in 20 mM K^+ ^^22^, and higher than that reported in lower external K^+^ (e.g. 0.1 mM in 0–2 mM K^+ ^^12,22^). Interestingly, the IC_50_ measured for both cisapride and terfenadine was similar in I663P channels to that measured in WT channels, despite I663P channels being trapped-open. This suggests that significant unbinding of these drugs does not occur during the test step −120 mV when the gate is trapped open. This is consistent with a previous observation that unbinding of MK-499, which is normally trapped behind the WT closed activation gate, from D540K channels, which open during hyperpolarization, occurs with a relatively slow time constant of >100 s^30^.

The recently reported hERG structure^14^ has provided significant insight to our understanding of the unique high affinity drug binding of the channel and provides the basis for how we might interpret the findings of the present study. The structure reveals a focussed region of electronegative potential at the top of the pore cavity and extended hydrophobic pockets which may accommodate drug molecules. The side chains of Y652 and F656 are located close to the entrance of these hydrophobic pockets suggesting that they may help to coordinate drugs directly, or modify access to, or the architecture of, the pockets themselves. The currently published structures do not contain densities describing the position of blocker drugs, nor do they reveal the influence of the S631A inactivation deficient mutation, which subtly alters the selectivity filter, on the shape of the hydrophobic pockets, or the position of Y652 and F656, which have previously been proposed to re-orient as a result of inactivation gating^11,38^. However, the structural data available are consistent with the presence of a binding site that could be allosterically modified by pore mutations or the permeant ion which may alter the electronegative focus, or the shape of, or access to, the drug binding pockets, such that a drug is more or less likely to coordinate. Evaluation of these possibilities is beyond the data presented in this study; however, solution of further high resolution structures in the presence of a drug in inactivation-intact and inactivation-removed channels promises to provide significant insight to the structural and molecular basis of drug binding.

## Material and Methods

### Molecular Biology

All hERG channel constructs were subcloned into the expression vector pBluescript SKII and expressed in *Xenopus laevis* oocytes. Conventional overlap extension PCR with primers synthesized by Sigma Genosys (Oakville, ON) were used for constructing hERG I663P and I663P/S620T mutant channels. The mutant channels were sequenced using Eurofins MWG Operon (Huntsville, AL) to ensure no errors were integrated during PCR cycling. Once the desired mutation was achieved, constructs were linearized using *XbaI* restriction endonuclease to create a template for *in vitro* transcription of cRNA. cRNA was transcribed from linear cDNA using the mMessagemMachine T7 Ultra cRNA transcription kit (Ambion, Austin, TX).

### Oocyte preparation and injection

Experimental protocols were approved by the Simon Fraser University Animal Care Committee (Protocol 1040K-08) and were in accordance with and Canadian Council on Animal Care. Oocytes were isolated from female *Xenopus laevis* frogs that were terminally anesthetised by immersion in 2 g/L tricaine solution (Sigma Aldrich) for 10–15 min. The ovarian lobes were surgically removed and partial digestion of follicular layers was achieved upon treatment with 1 mg/ml collagenase type 1A (Sigma Aldrich) in a calcium free solution (MgOR2) containing (in mM): 96 NaCl, 2 KCl, 20 MgCl_2_, 5 HEPES (titrated to pH 7.4) for 1 h. This was followed by manual removal of the remaining follicular layer of selected stage V-VI oocytes. Defolliculated oocytes were then injected with 50 nl (5–15 ng) of cRNA using a Drummond digital micro dispenser (Fisher Scientific, Nepean, Canada). After injection, oocytes were incubated in SOS+ medium containing (in mM): 96 NaCl, 2 KCl, 1.8 CaCl_2_, 1 MgCl_2_, 5 HEPES, 2.5 sodium pyruvate, 100 mg/L gentamycin sulfate and 5% horse serum (titrated to pH 7.4) at 19 °C for 2–5 days prior to electrophysiological recording.

### Data acquisition

Wild type (WT), I663P and I663P/S620T mutant hERG channel currents were recorded using conventional two-electrode voltage clamp with an OC-725C amplifier (Warner Instruments, Handen, CT). Signals were digitized using a digidata 1440 A/D convertor. Computer-driven protocols were performed using pClamp 10.2 software (Axon Instruments, Foster City, CA). Recordings were performed while the oocytes were continuously perfused with external ND96 containing 30 mM K^+^ (in mM: 69 NaCl, 30 KCl, 5 HEPES, 0.5 CaCl_2_, 1 MgCl_2_, titrated to pH 7.4) to augment inward current upon hyperpolarization and therefore achieve better resolution of drug block. Recording microelectrodes were made from borosilicate glass with a resistance of 0.2–2.0 MΩ when filled with 3 M KCl. Current signals were acquired at a sampling rate of 10 kHz and with a 4 kHz low-pass Bessel filter. Recordings were performed at 20–22 °C.

### Voltage protocols and data analysis

Data were analysed using Clampfit 10.3 (Axon Instruments) and SigmaPlot11 (Systat Software,San Jose, CA) software. In hERG I663P trapped-open mutant channels, inward current passed during the −80 mV holding potential. In these cases, a holding potential of −30 mV was used, since this correlated reasonably well with the reversal potential in 30 mM external [K^+^] solution. Rectification factor was calculated as previously described^1^ using: R = I/Gn(V − E_rev_), where R is the rectification factor, I is the membrane current, G is the slope conductance calculated from the fully activated current-voltage relationship, n is the activation variable (which was set at 1.0 since our data were collected from fully activated channels i.e. following a voltage step to +60 mV), V is the test voltage and E_rev_ is the measured reversal potential. The dependence of rectification factor upon voltage was then fitted with a Boltzmann function: y = 1/(1 + exp(*V*_*1/2*_ − V)/*k*), where *V*_*1/2*_ is the voltage of half-inactivation, V is the test voltage and *k* is the slope factor. The concentration-response relationship of drug interactions with WT and mutant hERG channels was described by the Hill equation: y = 1/(1 + (*IC*_50_/[drug])^*n*^), where y is the fractional conductance, *IC*_50_ is the concentration of the drug required to achieve half maximal block, [drug] is the concentration of the drug in question, and *n* is the Hill coefficient. While the holding potential used was −80 mV for WT channels and −30 mV for I663P channels (because this is close to the reversal potential of constitutively active I663P channels), this would not be expected to influence the measured IC_50_, since the extent of drug block was measured from peak inward current at −80 mV following a 500 ms step to +60 mV (to activate channels) in both channel types. The IC_50_ values yielded therefore represent channel availability in fully activated and inactivated channels, i.e., following a step to +60 mV, in both channels. To measure the extent of drug block in hERG I663P channels at −80, +40 or −40 mV, the membrane potential was clamped at either −80, +40 or −40 mV and repetitively stepped to −120 mV for 500 ms to measure the instantaneous tail current amplitude. Tail currents were measured in the absence of drug and the percentage block was measured once steady-state block was reached. This approach assumes that the rate of drug unbinding is slower than the speed of the voltage clamp, which is not unreasonable since the time course for the unbinding of cisapride from hERG channels has been reported to be in the order of minutes^31,34^. The approach also assumes that there is minimal unbinding from trapped-open channels during the −120 mV test step. While the unbinding rate of terfenadine and cisapride from I663P channels is not known, the unbinding time constant of MK-499 from D540K channels, which open during hyperpolarization was reported as 116 s at −120 mV^30^. This is significantly longer than the duration of the −120 mV test pulse used here, and therefore unbinding during the test step is anticipated to have minimal effect on the measured extent of channel block. Permeability ratios were calculated from current reversal potentials measured in external solutions (in mM: 5 HEPES, 0.5 CaCl_2_, 1 MgCl_2_, pH 7.4 using hydroxide of the major cation) containing either 99 mM LiCl, NaCl, KCl, RbCl or CsCl. Currents were recorded during 4 s voltage steps between −110 and 60 mV applied following a 500 ms step to +60 mV. Peak tail currents were plotted against voltage and the cubic polynomial fit, f = y_0_ + (ax) + (bx^2^) + (cx^3^), was used to determine the reversal potential. Permeability ratios (P_X_/P_K_) for each ion (X) were calculated relative to the permeability of K^+^ using: P_X_/P_K_ = exp((E_revX_ - E_revK_)/(RT/F)), where E_revX_ and E_revK_ are the measured reversal potentials in solutions containing 99 mM ion X^+^ or K^+^, respectively, R is the molar gas constant, T is the absolute temperature and F is Faraday’s constant. All data are presented as mean ± SEM (n = number of cells). Statistical tests using *t*-test and one-way or two-way analysis of variance (ANOVA) were used as appropriate (NS = not significantly different).

## References

[1] Sanguinetti MC, Jiang C, Curran ME, Keating MT (1995). A mechanistic link between an inherited and an acquird cardiac arrthytmia: HERG encodes the IKr potassium channel. Cell.

[2] Trudeau MC, Warmke JW, Ganetzky B, Robertson GA (1995). HERG, a human inward rectifier in the voltage-gated potassium channel family. Science.

[3] Sanguinetti MC, Tristani-Firouzi M (2006). hERG potassium channels and cardiac arrhythmia. Nature.

[4] Wang S, Liu S, Morales MJ, Strauss HC, Rasmusson RL (1997). A quantitative analysis of the activation and inactivation kinetics of HERG expressed in Xenopus oocytes. J Physiol.

[5] Curran ME (1995). A molecular basis for cardiac arrhythmia: HERG mutations cause long QT syndrome. Cell.

[6] Mitcheson JS, Chen J, Lin M, Culberson C, Sanguinetti MC (2000). A structural basis for drug-induced long QT syndrome. Proc. Natl. Acad. Sci..

[7] Mitcheson JS, Perry MD (2003). Molecular determinants of high-affinity drug binding to HERG channels. Curr. Opin. Drug Discov. Devel..

[8] Roden DM, Balser JR, George AL, Anderson ME (2002). Cardiac Ion Channels. Annu. Rev. Physiol..

[9] Raschi E, Vasina V, Poluzzi E, De Ponti F (2008). The hERG K+ channel: target and antitarget strategies in drug development. Pharmacol. Res..

[10] Kamiya K, Niwa R, Mitcheson JS, Sanguinetti MC (2006). Molecular Determinants of hERG Channel Block. Mol. Pharmacol..

[11] Chen J, Seebohm G, Sanguinetti MC (2002). Position of aromatic residues in the S6 domain, not inactivation, dictates cisapride sensitivity of HERG and eag potassium channels. Proc. Natl. Acad. Sci..

[12] Fernandez D, Ghanta A, Kauffman GW, Sanguinetti MC (2004). Physicochemical Features of the hERG Channel Drug Binding Site. J. Biol. Chem..

[13] Mitcheson JS (2008). hERG Potassium Channels and the Structural Basis of Drug-Induced Arrhythmias. Chem. Res. Toxicol..

[14] Wang W, MacKinnon R (2017). Cryo-EM Structure of the Open Human Ether-à-go-go -Related K+ Channel hERG. Cell.

[15] Ficker E, Jarolimek W, Kiehn J, Baumann A, Brown AM (1998). Molecular determinants of dofetilide block of HERG K+ channels. Circ. Res..

[16] Herzberg, I. M., Trudeau, M. C. & Robertson, G. A. Transfer of rapid inactivation and sensitivity to the class III antiarrhythmic drug E-4031 from HERG to M-eag channels. *J. Physiol*. 3–14, 10.1111/j.1469-7793.1998.003bi.x (1998). 10.1111/j.1469-7793.1998.003bi.x PMC2231109 9679158

[17] Weerapura M, Hébert TE, Nattel S (2002). Dofetilide block involves interactions with open and inactivated states of HERG channels. Pflugers Arch..

[18] Ficker E, Jarolimek W, Brown AM (2001). Molecular determinants of inactivation and dofetilide block in ether a-go-go (EAG) channels and EAG-related K(+) channels. Mol. Pharmacol..

[19] Perrin MJ, Kuchel PW, Campbell TJ, Vandenberg JI (2008). Drug Binding to the Inactivated State Is Necessary but Not Sufficient for High-Affinity Binding to Human Ether-a-go-go-Related Gene Channels. Mol. Pharmacol..

[20] Lees-Miller JP, Duan Y, Teng GQ, Duff HJ (2000). Molecular determinant of high-affinity dofetilide binding to HERG1 expressed in Xenopus oocytes: involvement of S6 sites. Mol. Pharmacol..

[21] McPate MJ, Duncan RS, Hancox JC, Witchel HJ (2008). Pharmacology of the short QT syndrome N588K-hERG K^+^ channel mutation: differential impact on selected class I and class III antiarrhythmic drugs. Br. J. Pharmacol..

[22] Barrows, B. *et al*. Extracellular potassium dependency of block of HERG by quinidine and cisapride is primarily determined by the permeant ion and not by inactivation. *Channels (Austin)*. **3**, 239–48 (2009). 19617705

[23] Wu W, Gardner A, Sanguinetti MC (2015). The Link between Inactivation and High-Affinity Block of hERG1 Channels. Mol. Pharmacol..

[24] Wang S, Morales MJ, Liu S, Strauss HC, Rasmusson RL (1997). Modulation of HERG affinity for E-4031 by [K+]o and C-type inactivation. FEBS Lett..

[25] Numaguchi, H. *et al*. Probing the Interaction Between Inactivation Gating and dd-Sotalol Block of HERG. *Circ. Res*. **87** (2000). 10.1161/01.res.87.11.1012 11090546

[26] Guo J, Gang H, Zhang S (2006). Molecular Determinants of Cocaine Block of Human Ether-a-go-go-Related Gene Potassium Channels. J. Pharmacol. Exp. Ther..

[27] Thouta S (2014). Proline scan of the hERG channel S6 helix reveals the location of the intracellular pore gate. Biophys. J..

[28] Kiehn J, Lacerda AE, Wible B, Brown AM (1996). Molecular physiology and pharmacology of HERG. Single-channel currents and block by dofetilide. Circulation.

[29] Spector, P. S., Curran, M. E., Keating, M. T. & Sanguinetti, M. C. Class III Antiarrhythmic Drugs Block HERG, a Human Cardiac Delayed Rectifier K+ Channel. *Circ. Res*. **78** (1996). 10.1161/01.res.78.3.499 8593709

[30] Mitcheson JS, Chen J, Sanguinetti MC (2000). Trapping of a methanesulfonanilide by closure of the HERG potassium channel activation gate. J. Gen. Physiol..

[31] Stork D (2007). State dependent dissociation of HERG channel inhibitors. Br. J. Pharmacol..

[32] Sanchez-Chapula JA, Navarro-Polanco RA, Culberson C, Chen J, Sanguinetti MC (2002). Molecular Determinants of Voltage-dependent Human Ether-a-Go-Go Related Gene (HERG) K+ Channel Block. J. Biol. Chem..

[33] Saxena P (2016). New potential binding determinant for hERG channel inhibitors. Sci. Rep..

[34] Kamiya K, Niwa R, Morishima M, Honjo H, Sanguinetti MC (2008). Molecular determinants of hERG channel block by terfenadine and cisapride. J. Pharmacol. Sci..

[35] Macdonald LC, Kim RY, Kurata HT, Fedida D (2018). Probing the molecular basis of hERG drug block with unnatural amino acids. Sci. Rep..

[36] Myokai T, Ryu S, Shimizu H, Oiki S (2008). Topological Mapping of the Asymmetric Drug Binding to the Human Ether-a-go-go-Related Gene Product (HERG) Potassium Channel by Use of Tandem Dimers. Mol. Pharmacol..

[37] Yang B (2004). Inactivation gating determines drug potency: a common mechanism for drug blockade of HERG channels. Acta Pharmacol. Sin..

[38] Lin J (2005). Intracellular K+ is required for the inactivation-induced high-affinity binding of cisapride to HERG channels. Mol. Pharmacol..

[39] Perry MD, Wong S, Ng CA, Vandenberg JI (2013). Hydrophobic interactions between the voltage sensor and pore mediate inactivation in Kv11.1 channels. J. Gen. Physiol..

[40] Pareja K, Chu E, Dodyk K, Richter K, Miller A (2013). Role of the activation gate in determining the extracellular potassium dependency of block of HERG by trapped drugs. Channels (Austin)..

